# Teeth Damage during General Anesthesia

**DOI:** 10.3390/jcm12165343

**Published:** 2023-08-17

**Authors:** João M. Neto, Ana Rita Teles, Joselina Barbosa, Orquídea Santos

**Affiliations:** 1 School of Dentistry, University Institute of Health Sciences (IUCS), Cooperativa de Ensino Superior Politécnico e Universitário (CESPU), 4585-116 Gandra PRD, Portugal; orquidea.santos@iucs.cespu.pt; 2 Department of Anesthesiology, Centro Hospitalar Universitário de São João, 4200-319 Porto, Portugal; 3 Faculdade de Medicina, Universidade do Porto, 4169-007 Porto, Portugal; joselina@med.up.pt

**Keywords:** oral damage, dental injury, laryngoscopy, anesthesia, general anesthesia

## Abstract

Introduction: Dental injuries during anesthesia, especially when advanced airway management is required, represent a legal problem. Factors such as poor dental condition and excessive pressure during intubation contribute to dental damage. The maxillary central incisors are commonly affected. Objective: The objective of this review is to know the incidence of dental injuries in adults undergoing anesthesia that requires airway management. Materials and Methods: The search was performed in MEDLINE (through Pubmed), ClinicalTrials.gov, Scopus, LILACS (through the Virtual Health Library Regional Portal), and SciELO for all available literature on the subject up to December 2022. Inclusion criteria involved articles that studied patients aged 18 years or older who underwent general anesthesia requiring airway management with tracheal intubation or insertion of a laryngeal mask airway. Results: Of all the articles, nine report dental injury associated with the type of airway management. Only one article does not have dental injury. Discussion: This study addresses dental injuries related to tracheal intubation during general anesthesia. Although techniques are used to prevent them, these injuries still occur. Laryngoscopy, especially with support on the upper central incisors, can cause damage to the teeth. Conclusions: It is important that the anesthesiologist is aware of dental trauma and that orotracheal intubation or the placement of the laryngeal mask airway is performed systematically and rigorously, always considering the patient’s dentition to choose the best approach in each specific situation.

## 1. Introduction

Perioperative dental injury is the most common complaint among all medico-legal complaints related to anesthesia and is the cause of one-third of the lawsuits regarding medico-legal anesthesia [1].

Dental trauma or, as a matter of fact, any intra-oral trauma caused as a result of anesthetic practice is a relevant issue which, apart from playing a contributing role to the overall morbidity of a patient, can also lead to litigation issues [2].

It is important to point out that these injuries occur during surgical procedures unrelated to pre-existing dental trauma. It can be detrimental to the patient’s well-being, especially when the patient should not expect complications such as pain, aesthetic, and functional problems resulting from dental trauma which significantly disrupt normal function and quality of life [3]. In addition, the cost of replacing damaged or lost teeth can be significant [4,5]. In a few extreme cases, even near-fatal complications, such as esophageal perforation and mediastinitis following aspiration of a dental prosthesis or a tooth, have been described.

In the peri-operative period, the majority of dental injuries (50–75%) occur during tracheal intubation [1]. The overall incidence of dental injury is estimated to be between 0.06% and 12%, but these values may be underestimated [6].

The incidence of dental damage during orotracheal intubation depends on several factors such as poor dental conditions, significant pressure on the dental arch, and misuse of the protective device supplied by the hospital is often a contributing factor [7]. Also, when a satisfactory view of the glottis is difficult to obtain during laryngoscopy, the patient’s maxillary anterior teeth are sometimes used as a fulcrum by the laryngoscope blade [8]. The majority of these occurrences are caused by the application of pressure from the hard metallic blade of the laryngoscope [6,9,10]. There are three major groups of causative factors: unfavorable patient anatomy, the experience and skill of the anesthesiologist who handles the airway, and the design of the laryngoscope blade [8]. In the study of Diakonoff et al., that review 21 years of law decisions, he found poor dentition in 15 of 19 cases, 78.9% preoperatively document, pre-existing periodontal disease in 16 cases (66.7%), and dental restorations or prostheses in seven cases (30.4%) [11]. Dental injury occurred in 21 cases (87.5%) where intubation was performed [11].

In cases of emergent airway management, the incidence of oral trauma increases to 7% [8]. The most common dental injuries in these cases include enamel fractures, loosened, subluxated teeth, tooth avulsion, crown or root fractures, luxation, and missing teeth [2,6,8,9,10].

The anterior sextant of the maxillary region, more specifically the central maxillary incisors, are the most affected [9]. In fact, the left central incisor is reported to be the tooth with the highest risk of dental injury, due to its direct contact with the laryngoscope blade as well as its use as a fulcrum to position the laryngoscope [9]. However, there are few publications on registration strategies that support the implementation of effective preoperative measures to prevent damage, namely through the laryngoscopy procedures [9]. Nor is this damage a commonly discussed problem in the literature, although it is a subject which the Medical Protection Society warns its members about in a special publication [12].

Protective devices, such as mouthguards, are recommended to prevent dental injuries caused by poor dentition [13]. Although the use of mouthguards has been documented to reduce the force inflicted upon the anterior dentition during laryngoscopy, the effectiveness of a mouthguard as a preventive tool against dental injuries remains controversial, and even when indicated it is rarely used [11,13].

Considering the magnitude of the problem and its physical, economic, and legal consequences, it is important to determine the risk factors, frequency, and outcomes of dental injuries related to anesthesia [1]. Furthermore, raising awareness among anesthesiologists about the significance of this problem is crucial [6]. They should be educated about tooth anatomy, supporting structures, common dental pathologies, and the techniques used in dental rehabilitation to effectively address and prevent dental injuries related to anesthesia [9,10].

The objective of this review is to know the incidence of dental injuries in adults who underwent anesthesia that required airway management and as a second aim to characterize the type of dental damage that is related to the type of device used to approach the airways.

## 2. Materials and Methods

The protocol of this systematic review and meta-analysis was written following the PRISMA-P (Preferred Reporting. Items for Systematic Review and Meta-Analysis Protocols) guidance.

### 2.1. Pico

#### 2.1.1. Patient, Population, or Problem

- Adult patients undergoing general anesthesia and teeth damage.

#### 2.1.2. Intervention or Exposure

- To see how many times patients got injured with the air way method.

#### 2.1.3. Comparison

- Intubation vs. Insertion supraglottic device.

#### 2.1.4. Outcome

- Frequency of oral injury.

### 2.2. Research Strategy

A search was conducted on MEDLINE (through Pubmed), ClinicalTrials.gov, Scopus, LILACS (through Virtual Health Library Regional Portal), and SciELO for all available literature on the subject until December 2022, using the following Mesh Terms: [(Dental injury) AND (Laryngoscopy) AND (Anesthesia)] or [(Dental injury) AND (Laryngoscopy) AND (Anesthesia) AND (General Anesthesia)].

The queries evaluated were: Types of injuries in our oral health; Risk factors; Types of laryngoscope; Solutions to minimize the problem.

### 2.3. Eligibility Criteria

Inclusion Criteria: papers that studied dental damage in patients with 18 years of age or older that underwent general anesthesia requiring airway management with tracheal intubation or laryngeal mask insertion.

Exclusion criteria comprised pediatric patients, animals or phantom studies, ongoing studies, lack of study measures, patients who underwent oral surgical procedures, case reports, review articles and papers that are not in Portuguese or English.

### 2.4. Study Selection and Data Collection

Firstly, the articles were selected based on the title and abstract according to the eligibility criteria. Then, the complete articles were obtained and read in full by the two authors, and it was decided whether they met the requirements of the eligibility criteria. Uncertainty and disagreement were resolved via discussion between the reviewers. The data extraction from the selected studies was independent and duplicate to avoid errors and reduce biases. Any remaining discrepancies were be resolved by a third author.

From each eligible study, we collected information about the study (year of publication, study time, country, type of study), on patient baseline demographics (age and sex distribution), cause for airway management (urgent or elective), airway management (tracheal intubation or supraglottic device), pre-operative dental assessment, frequency of dental damage, and type of dental injuries.

### 2.5. Risk of Bias in Included Studies

The quality of studies included in this systematic review was scored by two researchers using the Newcastle Ottawa Scale (NOS) (with a score ranging from 0 to 9 points) [14]. The NOS is a review tool for evaluating risk of bias in observational studies. The scale consists of four domains of risk of bias assessment; (i) selection bias; (ii) performance bias; (iii) detection bias, and (iv) information bias.

### 2.6. Synthesis of Evidence

Because of the heterogeneity of the included studies no meta-analysis was conducted in the present review. Regarding oral injuries incidences, we have used proportions and corresponding 95% confidence intervals (CIs) or the raw data that could be used to calculate the estimate.

## 3. Results

### 3.1. Search Strategy

**Figure 1:**
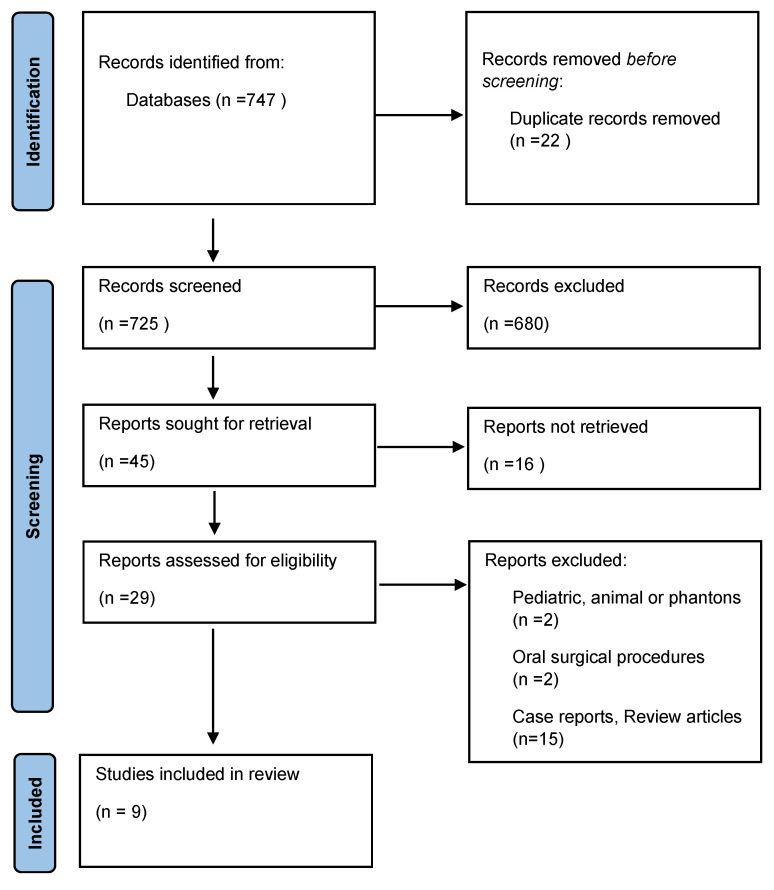
Prisma Flow chart outlining the selection of studies for review.

A total of 747 records were identified from databases, 22 duplicates were removed. The duplicate studies were removed after reading the titles and the authors of each study by one investigator. The remaining 725 articles were submitted to abstract screening by two researchers, and 680 were excluded. The last 45 reports were reviewed in full and after the application of the inclusion and exclusion criteria, 29 studies were selected for final analysis. Of these 29 selected studies, 15 were excluded for not addressing the topic, 2 for having oral surgical procedures, and another 2 for addressing studies in pediatrics, animals or phantoms, leaving in the end only 9 articles (Figure 1).

**Figure 2:**
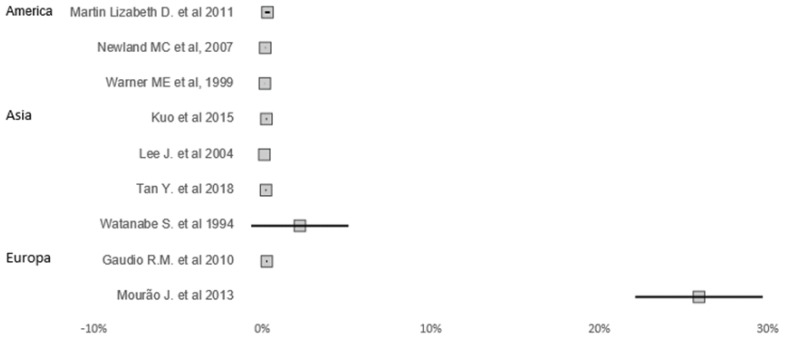
Prevalence and 95% confidence interval of studies providing information of dental injuries during airway management [15,16,17,18,19,20,21,22,23].

In all studies, it is possible to verify that there are dental injuries during the intubation process. Studies were conducted in Europe, America, and Asia, as seen in Figure 2.

As seem in Table 1, the frequency of dental injury was almost absent. One exception was a study in Europe which found a prevalence of 25% (95% CI: 21%, 29%) [15].

Therefore, Newland et al. analyzed 78 dental injuries in 161,687 patients (0.048%; 95% CI: 0.04%, 0.06%) from adverse event database records from 1989 to 2003 in which 86% were discovered by the anesthetist, and 14% by the patient [16]. Warner et al. reported 132 dental injuries in 486,791 (0.027%; 95% CI: 0.02%, 0.03%) anesthetic procedures reported by patients 7 days after the procedure [17]. Martin et al. analyzed six dental injuries in 3423 patients (0.175%; 95% CI: 0.04%, 0.32%) that made a complain between the years 2001 and 2009 [18]. Tan et al. analyzed 51 dental injuries in 55,158 patients (0.092%; 95% CI: 0.07%, 0.12%) of an audit data base from 2011 to 2014 in which 44 (86.3%) were discovered in the operating theatre, 4 (7.8%) were discovered in the post-anesthetic care unite, and 1 (2%) in the ward [19]. Mourão et al. reported 134 dental injuries in 536 patients 25% (95% CI: 21%, 29%) that underwent laryngoscopy or in which all injuries were discovered by a health professional between 12 and 36 h after the anesthesia procedure [15]. Lee et al. in their clinical trial did not report any injuries [20]. Watanabe et al. sustained two dental injuries during 382 laryngoscopies in 98 patients of 2.041% (95% CI: 0.008%, 4.8%) performed by the same senior anesthesiologist, which performs an average incidence of 1/191 of dental injuries vs. laryngoscopy [21]. Kuo et al. analyzed 76 dental injuries 64,718 patients of 0.117% (95% CI: 0.09%, 0.14%) of adverse events data base records from 2010 to 2011 [22]. Gaudio et al. analyzed 83 dental injuries in 60,000 patients of 0.138% (95% CI: 0.11%, 0.17%) that made a complain between the years 2000 and 2008 [23].

### 3.2. Types of Tooth Injury

The types of tooth injury were only reported by six articles.

Newland et al., reported eight types of injuries that affected 78 patients described from dental consultation: 25 (32.1%) enamel fractures; 18 (23.1%) loosening/subluxations; 3 (3.8%) luxations; 7 (9%) avulsions; 6 (7.7%) Crown fractures; 1 (1.3%) crown and root fracture; 8 (10.3%) missing tooth/teeth; 17 (21.8%) other minor complications [16]. Tan et al. only reported that in 3 patients out of 51 who suffered injuries there were two cases (66%) of tooth avulsion and 1 case (34%) of crown fracture diagnosis by the dentistry of the hospital [19]. Mourão et al. reported two types of injuries that affected 86 patients: 82 (15%) enamel fractures; 4 (0.7%) avulsions using the modified WHO’s classification [15]. Watanabe demonstrated 1 patient out of 2 affected patients had a subluxation and another had a crown fracture, then treated by the institutional dentistry [21]. Kuo et al. reported seven types of injuries from 76 patients who suffered an injury, identified by anesthetist, patients, or nurses: 8 (20%) loosening/subluxations; 2 (5%) dislocations; 14 (35%) avulsions; 6 (15%) coronary fractures; 1 (2.5%) missing tooth/teeth; 4 (10%) other minor complications; 5 (12.5%) fixed partial denture damage [22]. Gaudio et al. reported five types of injuries that 83 patients suffered: 1% enamel fracture; 3.8% dislocation; 50% Avulsion; 14% coronary fracture; 12% crown and root fracture [23].

### 3.3. Types of Airways Management and Teeth Damage

As seen in Table 1, different forms of airway management were performed, however not all of them affected the teeth. Newland et al. reports that of the 78 dental injuries, 75 were due to Laryngoscopy; 2 due to facial Masks, and 1 due LMA [16]. Warner et al. demonstrate that the 132 dental injuries were caused by laryngoscopy [17]. Martin et al. demonstrate that the six dental injuries were caused by Laryngoscopy [18]. Tan et al. reports that of the 51 dental injuries, 40 dental injuries were due to Laryngoscopy, 7 dental injuries were due to SADs, 3 to double lumen tube, and 1 dental injury due to a mask. Of those with descriptive data, laryngoscopy was the most common cause of dental injury [19]. Mourão et al. demonstrate that the 134 dental injuries were due to Laryngoscopy [15]. Watanabe et al. demonstrate that the two dental injuries were due to Laryngoscopy [21]. Kuo et al. demonstrate that 42.1% of the dental injuries were due to LMA and 28.9% were due to Laryngoscopy [22]. Gaudio et al. demonstrate that the 83 dental injuries were due to Laryngoscopy [23].

### 3.4. Teeth Affected

In general, it is possible to verify in almost all articles that the most affected teeth are the upper central incisors.

Newland et al. reports that the upper left and right central incisors were the most affected. From the right lateral incisor to the right first premolar moderately affected. Left lateral incisor to left first premolar moderately affected [16]. Warner et al. reports that the teeth the most affected were the upper central incisors [17]. Tan et al. reports that upper right and left incisors were the most affected, although there were also three injuries to the lower left central incisor, one injury to the right upper canine and right upper premolar, one injury to the lower right central incisor and right lower canine and one injury on the left lower canine and left premolar [19]. Mourão et al. reports 80 injuries to the upper right central incisor, 3 injuries to the upper right lateral incisor, 53 injuries to the upper left central incisor, 11 injuries to the upper left lateral incisor, 4 injuries to the lower left central incisor, 1 injury to the lower left lateral incisor, 8 injuries to the lower right central incisor, 1 injury to the lower right lateral incisor, and 1 injury to the lower right canine [15]. Watanabe et al. reports that the most teeth affected were the upper central incisors [21]. Kuo et al. reports that there were 15 lesions in the left central incisor; 9 injuries to the right central incisor; 3 injuries to the right lateral incisor; 5 injuries to the left lateral incisor; 4 injuries to the lower left central incisor; 2 injuries to the lower left lateral incisor [22]. Gaudio et al. reports that in their study 90% the teeth affected were upper incisors [23].

## 4. Discussion

The outcome was difficult to obtain because three studies were prospective, and five studies based on data collected from claims or audits or reports from professionals involved in the patient’s treatment.

Although this is an event that occurs less than two in a hundred patients, according to most of the included studies, they are common complications of general anesthesia and account for a significant proportion of all medicolegal claims against anesthesiologists, especially in France, where they represent 40% of these claims [11,16,24].

In the results, it is possible to verify that Warner et al. and Mourão et al. are the ones with the highest number of lesions, as they were prospective studies [15,17]. These two studies have the highest number of lesions due to the fact that the analysis of the oral cavity is always perform 7 days and 12 to 36 h after anesthesia, respectively. While the other studies only analyzed the database of patients or anesthesiologists or nurses who reported an incident, there is no certainty whether more injuries occurred or not.

The incidence of dental damage during airway management depends on several factors; a preexisting poor dentition with large decays or restorations, advanced periodontitis, presence of dental prosthesis, shedding primary teeth, jaw misalignment, anterior crowding are well-recognized risk factors and difficulties in laryngoscopy, in these cases anesthesiologists are apt to rotate the laryngoscope handle even further posteriorly. This often causes direct contact with the upper teeth, which are then used as a fulcrum for the posterior heel or horizontal flange [20,21,22].

Older studies, such as Newland et al., report that the most frequent injury is enamel fracture [16]. However, in our revision it was possible to verify that avulsion is one of the biggest injuries that occurred in oral health as in the study of Diakonoff et al., that reviewed 21 years of judicial decisions from peri-anesthetic dental injuries [11].

In Gaudio et al., as demonstrated in their study, 50% of the injuries were avulsions, putting the hypothesis that this is due to mobility, since most of the avulsions occurred during “smooth” procedures of laryngoscope maneuvers [23]. Kuo et al. also reported in their study that 35% of the injuries were avulsions, with a decrease occurring because they did not use direct laryngoscopy in teeth with mobility [22]. Mourão et al. and Kuo et al. are the only prospective studies, where an analysis of the preoperative and one postoperative oral cavity was carried out, and it is possible to verify that the most frequent lesion of Mourão et al. were enamel fractures [15,22]. Although both these authors performed a pre-operative dental assessment, they could not find an association between the preoperative dental sate and dental injury, which is different from the retrospective or insurance studies [1,7,11,12,16,17,19,23,25].

As reported by several previous studies, the results show that the most affected teeth are the upper central incisors, this is because routine laryngoscopy exerts great forces on the maxillary teeth, and the prominent flange of the Macintosh blade may contribute [15]. Also most anesthesiologists use the maxillary incisors as a fulcrum, so when there is a bad visibility of supraglottic, they are guided by the upper central incisors, often causing injuries [15]. However, it was possible to verify that the upper lateral incisors, especially the left lateral incisor, and the lower incisors demonstrate a high probability of presenting injury. Mourão et al., in comparison with other studies, presented a greater variety of affected teeth, demonstrating that the upper and anterior teeth have a greater risk of presenting lesions, and it is possible to demonstrate that the right side of the oral cavity is the most affected [15].

Watanabe et al. and Lee et al. studied the distance between the laryngoscope blade and the teeth, verified in each study that a low-heeled blade could allow a better view of the supraglottic between the blade and the upper teeth and could reduce the incidence of dental injury [20,21].

In the analyzed studies, the tracheal intubation with a laryngoscopy was considered a leading cause of dental injury, particularly when a glottis view was difficult to obtain [22]. In our study, it is possible to observe that laryngoscopy in most of the studies is the cause of dental injury, although it was possible to verify, in comparison with the analyzed studies, that only three studies used LMA and that in one of these three studies there was a higher number of injuries associated with LMA compared to laryngoscopy.

Burton et al., in their study, refer that only 9% of the anesthetists routinely took precautions for all their laryngoscopies. A greater proportion (50%) claimed to routinely use teeth protection for patients considered to be at risk. So, despite the belief that the incidence was high, there were a large number of anesthetists who did not worry unduly about precautions. One of the reasons that this occurs is that many anesthetists believe that the protection methods, with the exception of tongue-type laryngoscope blades, make access through the mouth more difficult by adding extra material over the upper teeth [12]. In addition, it was possible to observe in two studies a concern that is not yet very evident in this type of study, the cost of each repair. The first to realize this concern was Warner et al. (who did not obtain the costs of 19% of the injured), who had an estimate of 88–8500 for each repair [17]. Gaudio et al., demonstrated that all repairs for the 83 patients cost between EUR 200–3500, with a total cost of EUR 85,000 [23]. The high costs associated with repairing injuries greatly increase the likelihood the patient will pursue a claim. Partial or total payment was provided to all 83 patients regardless of evident preoperative pathology. If all charts contained appropriate documentation and patients were adequately informed on the existence of a high risk of dental trauma, when present, the amount of reimbursement might have been lower [23].

As a solution to minimize dental injuries, it is suggested that a complete oral examination should be performed in order to decrease injuries caused by the anesthesiologist; various types of blades to be used as a rubber laryngoscope would have less impact on the teeth, and could be used as a way to train a trainee anesthesiologist; a Belscope blade, as it is more practical and reduces the possibility of direct contact with the upper teeth; a modified low-height flange on Macintosh blade would reduce the frequency of direct contact between the blade and the upper teeth by over 80%, and a plastic guard should be used to protect the teeth [15,16,20,21,23]. Also, after a complete examination by the anesthesiologist, patients should be forwarded to dentists to treat teeth at high risk of damage (that is, with old restorations or critical periodontal conditions) and/or proceed with a custom-made protective splint for the patient to wear prior to the endotracheal intubation, which would significantly reduce the risk of dental damage, or the protection of teeth during tracheal intubation procedures can be achieved by using an intraoral scan of the patient and a custom-made, 3D-printed mouthguard [7].

## 5. Conclusions

It is demonstrated that airway management is still a legal issue for dental trauma.

Avulsion still continues to be one of the most common dental injuries during general anesthesia associated with laryngoscopy for patients with advanced periodontitis or shedding primary teeth.

The limitation of this study has to do with the heterogeneity of the methodology of included studies, and it was possible to observe that all studies had similar results regarding the most affected teeth and the type of airway management that caused it.

In the future, a randomized observational study should be carried out comparing these three types of devices in order to reach a conclusion as to which technique causes more dental injuries.

## Figures and Tables

**Table 1:** Results.

Author, Year of Publication	Title	Aim of the Study	Type of Airway Management	Total Quality Score	Types of Tooth Injury	Most Tooth Affected	Conclusions
Newland MC et al., 2007 [8]	Dental injury associated with anesthesia: a report of 161,687 anesthetics given over 14 years	Determine the frequency, outcomes, and risk factors for dental injury related to anesthesia.	Mask only; Oral intubation; Nasal intubation; LMA; Tracheostomy	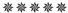	Enamel fracture; Subluxation; Luxation; Avulsion; Crown Fracture; Crown and root fracture; Missing tooth/teeth; Other injury	Upper central incisors	Dental injury is one of the most common adverse events reported in association with anesthesia. Risk factors include preexisting poor dentition or reconstructive work and moderately difficult to difficult intubation [8].
Warner ME et al., 1999 [9]	Perianesthetic dental injuries: frequency, outcomes, and risk factors	Determine the frequency, outcomes, and risk factors for perianesthetic dental injuries	Laryngoscopy, tracheal intubation	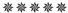	Crown fractures, Partial dislocations	Upper incisors	Based on these data from a large surgical population at a single training institution, approximately 1:4500 patients who receive anesthesia services sustain a dental injury that required repair or extraction. Patients most at risk for perianesthetic dental injury include those with preexisting poor dentition who have one or more risk factors for difficult laryngoscopy and tracheal intubation [9].
Martin Lizabeth D. et al., 2011 [10]	3423 Emergency Tracheal Intubations at a University Hospital Airway Outcomes and Complications	Evaluate the incidence of difficult intubation and complication rates and to determine predictors of complications in this setting	Aspiration, Esophageal intubation, and Oropharyngeal	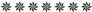			During emergent nonoperative intubation, specific clinical situations are associated with an increased risk of airway complication and may provide a starting point for allocation of experienced first responders [10].
Tan Y. et al., 2018 [11]	Dental injury in anesthesia: a tertiary hospital’s experience	Evaluate the incidence, risk factors, and local practices in the management of perioperative dental injuries in Singapore	three types of Supraglottic airway devices (SADs): all videolaryngoscopy was carried out using the McGrath Mac laryngoscopy	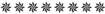	Avulsion and Crown Fractured	Upper right and left incisors	Videolaryngoscopy with the McGrath MAC is associated with an increased likelihood of dental injury. This could be either because videolarygoscopes were used when increased risk of dental trauma was anticipated, or due to incorrect technique of laryngoscopy. Future studies should be performed to establish the causality. The management of dental injuries could be improved with development of departmental guidelines [11].
Mourão J. et al., 2013 [12]	Dental injury after conventional direct laryngoscopy: a prospective observational study	Overcome some of the previous limitations and determine a more accurate incidence of dental damage and risk factors after conventional direct laryngoscopy for tracheal intubation	Laryngoscopy for tracheal intubation	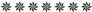	Enamel Fracture, Avulsed	Upper central incisor	Finally, given the high rate of reported injury with conventional laryngoscopy, it would be important to repeat our methodology using other intubation devices, such as videolaryngoscopes, to assess if the rate of injury is different [12].
Lee J. et al., 2004 [13]	The Callander laryngoscope blade modification is associated with a decreased risk of dental contact	Determine whether preoperative examination could predict the risk of contacting the teeth with the laryngoscope and to evaluate the effectiveness of a modified Macintosh blade on reducing dental 7 contact	Laryngoscopy	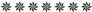			Airway characteristics correlate with the risk of hitting the upper teeth during laryngoscopy. The modified Macintosh blade reduces the risk of contacting the teeth [13].
Watanabe S. et al., 1994 [14]	Determination of the distance Between the Laryngoscope Blade and the Upper Incisors During Direct Laryngoscopy: Comparisons of a Curved, an Angulated Straight, and Two Straight Blades	Compare the heel-tooth distance when the optimum visibility of the glottis was obtained using the four different types of laryngoscope blade: a Miller, a Wisconsin with a higher heel than a Miller, a Macintosh, and a Belscope blade	Laryngoscopy	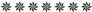	Fracture and Subluxation	Central incisor	In conclusion, a low-heeled angulated straight blade, the Belscope blade, provides a significantly greater field of view between the posterior end of the blade and the upper teeth than other types of blades. It may therefore contribute to a reduced likelihood of upper dental injuries during laryngoscopy [14].
Kuo et al., 2015 [15]	Quality improvement program reduces perioperative dental injuries e A review of 64,718 anesthetic patients	Reduce the incidence of perioperative dental injury.	LMA	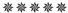	Subluxation; Luxation; Avulsion; Crown Fracture; Damage to fixed partial denture, Missing tooth, other injury	Upper central incisor	Dental injury incidence was significantly reduced and remained at low levels after implementation of the quality improvement program. We suggest the implementation of a standardized dental examination into the preoperative evaluation system adding pathologic teeth fixed or protected devices to minimize dental injury associated with anesthesia [15].
Gaudio R.M. et al., 2010 [16]	Traumatic dental injuries during anesthesia: part I: clinical evaluation	Investigate the main characteristics of the dental injuries identified by an anesthesiology incident reporting and constituting a body of malpractice claims.	Tracheal intubation	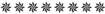	Avulsions, damage to crowns and bridgeworks, Luxation, Teeth fractures, Bulk fractures	Upper central incisor	Even though the majority of anesthesiologists were trained enough in the use of airway devices and aware of the potential damage while using excessive forces, some unexpected difficulties may have led to lesions. It is known that damage to teeth can occur even in the absence of negligence [16].

## Data Availability

The data that support the findings of this study are available from the corresponding author, J.N, upon reasonable request.
